# A Causal Web between Chronotype and Metabolic Health Traits

**DOI:** 10.3390/genes12071029

**Published:** 2021-07-01

**Authors:** John A. Williams, Dominic Russ, Laura Bravo-Merodio, Victor Roth Cardoso, Samantha C. Pendleton, Furqan Aziz, Animesh Acharjee, Georgios V. Gkoutos

**Affiliations:** 1 Centre for Computational Biology, Institute of Cancer and Genomic Sciences, University of Birmingham, Birmingham B15 2TT, UK; drr719@student.bham.ac.uk (D.R.); lxb732@student.bham.ac.uk (L.B.-M.); V.RothCardoso@bham.ac.uk (V.R.C.); scp887@student.bham.ac.uk (S.C.P.); f.aziz@bham.ac.uk (F.A.); a.acharjee@bham.ac.uk (A.A.); g.gkoutos@bham.ac.uk (G.V.G.); 2 Institute of Translational Medicine, University of Birmingham, Birmingham B15 2TT, UK; 3 NIHR Surgical Reconstruction and Microbiology Research Centre, University Hospital Birmingham, Birmingham B15 2WB, UK; 4 MRC Health Data Research UK (HDR), Midlands Site, Birmingham B15 2TT, UK; 5 NIHR Experimental Cancer Medicine Centre, Birmingham B15 2TT, UK; 6 NIHR Biomedical Research Centre, University Hospital Birmingham, Birmingham B15 2WB, UK

**Keywords:** circadian rhythm, chronotype, diabetes, alcohol intake, bipolar disorder, mendelian randomization

## Abstract

Observational and experimental evidence has linked chronotype to both psychological and cardiometabolic traits. Recent Mendelian randomization (MR) studies have investigated direct links between chronotype and several of these traits, often in isolation of outside potential mediating or moderating traits. We mined the EpiGraphDB MR database for calculated chronotype–trait associations (*p*-value < 5 × 10^−8^). We then re-analyzed those relevant to metabolic or mental health and investigated for statistical evidence of horizontal pleiotropy. Analyses passing multiple testing correction were then investigated for confounders, colliders, intermediates, and reverse intermediates using the EpiGraphDB database, creating multiple chronotype–trait interactions among each of the the traits studied. We revealed 10 significant chronotype–exposure associations (false discovery rate < 0.05) exposed to 111 potential previously known confounders, 52 intermediates, 18 reverse intermediates, and 31 colliders. Chronotype–lipid causal associations collided with treatment and diabetes effects; chronotype–bipolar associations were mediated by breast cancer; and chronotype–alcohol intake associations were impacted by confounders and intermediate variables including known zeitgebers and molecular traits. We have reported the influence of chronotype on several cardiometabolic and behavioural traits, and identified potential confounding variables not reported on in studies while discovering new associations to drugs and disease.

## 1. Introduction

With the advent of large biobank cohorts to provide genome-wide association study (GWAS), there have been several recent studies looking at the causal influence between chronotype (morningness or eveningness) exposures and both neurobehavioral/psychiatric- and cardiometabolic-related outcomes. Lind and colleagues found significant genetic correlations between oversleeping, insomnia, and undersleeping exposures with an outcome of post-traumatic stress disorder [1], but when testing for causality did not find evidence for causal effects of sleep phenotypes on post-traumatic stress. Adams and Neuhausen were interested in the interplay between chronotype and free fatty acid circulation, as well as potential associations between free fatty acid circulation and type 2 diabetes [2]. So as to evaluate this, they conducted two Mendelian randomization (MR) studies using two-sample data, and found that morning chronotype is associated with lower total fatty acid levels (inverse variance-weighted estimator [IVW] β−0.21, *p* = 0.02) and that elevated fatty acid levels are associated with a decrease in diabetes, granting a protective effect (IVW β−0.23, *p* = 0.01). They then extended their analysis to include subtypes of free fatty acids and their conclusions held, indicating that a morning chronotype is associated with lower mono-unsaturated fatty acid intake. Richmond and colleagues sought to model sleep traits and risk of breast cancer using chronotype, sleep duration, and insomnia GWAS for instrumental variable selection [3]. They showed a morning chronotype to be protective against breast cancer (odds ratio [OR] 0.85). Additional analyses supported these findings, showing a morning chronotype (IVW OR 0.88) to be protective against breast cancer, while increased sleep duration had a detrimental effect (IVW OR 1.19). Gibson investigated bi-directional causal effects of smoking on sleep duration and chronotype [4]. They found no clear evidence that smoking initiation influenced sleep behaviors directly, nor evidence for causal effects between chronotype on smoking behavior. However, they did find evidence that insomnia could lead to an increase in smoking behavior (IVW β 1.21, *p* = 0.02) in an under-powered analysis. Treur modelled caffeine consumption and sleep traits, including chronotype, sleep duration, and history of insomnia [5]. While the association between caffeine consumption and disturbed sleep is well known, their analysis did also show strong genetic correlations between those traits; however, their MR analyses failed to produce significant causal associations. At a wider scale, Lane and colleagues used MR analysis as a follow up to their first GWAS of chronotype using the UK Biobank [6]. They found significant associations between evening phenotype and years of education increasing and self-reported schizophrenia diagnosis, and associations between a morning chronotype and a decreased body mass index.

These studies, each taken in isolation, paint a compelling picture of relationships between chronotype and metabolic traits. However, *p*-values reported were generally >0.001. If analyzed as a whole set of experiments under multiple testing, results would often fail to achieve statistical significance.

Additionally, most studies fail to address confounding factors which may be identifiable by performing MR tests between exposures and potential confounders. In concurrence with these studies being conducted, large databases of GWAS and MR studies have been built, including the GWAS Catalog [7] and MR-Base [8]. In this study, we have taken advantage of GWAS study statistics and MR analyses surrounding chronotype to identify causal relationships between exposure to chronotype and several cardiometabolic- and mental health-related traits. We have additionally mined MR repositories for potential confounding factors, identifying both non-detrimental intermediate analyses (explanatory mediators and reverse mediators) and confounders which will need to be controlled for in mechanistic studies used to validate MR experiments.

## 2. Materials and Methods

**Figure 1:**
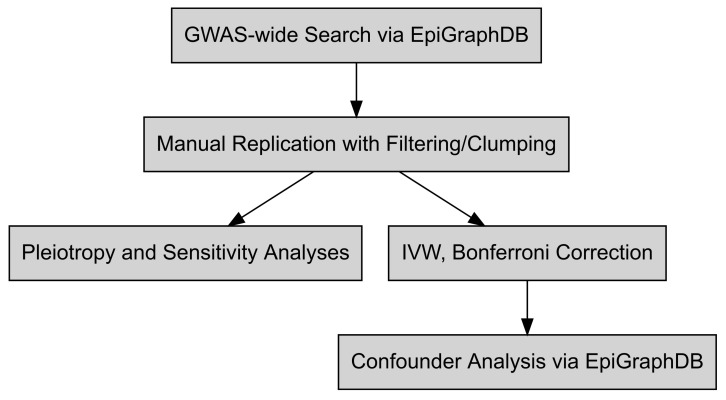
Mendelian randomization (MR) workflow for testing the causal influence of chronotype on traits in the EpiGraphDB database. An unbiased search for GWAS-derived two-sample MR studies was performed with chronotype or moningness/eveningness as reported exposures, and IVW results (*p* < 5 × 10^−8^) retained. Potential chronotype IVs were processed with Steiger filtering, LD clumping, and stand/palindromic SNP harmonization. IVW and related MR analyses are then performed on filtered SNP summary statistics, followed by pleiotropy (MR-Egger) and leave-one-out sensitivity analyses. Associations that survive multiple testing correction are then uploaded to EpiGraphDB to investigate potential confounders not present in exposure/trait analyses as reported.

A general workflow for this study is depicted in Figure 1. Using the EpiGraphDB R API v1.0 [9], results of pre-computed Mendelian randomization studies were obtained with the following parameters: exposure trait either “Morning/evening person (chronotype)” or “Chronotype”, with a *p*-value threshold of 5 × 10^−8^ (GWAS genome-wide significance). Results were filtered to significant random-effects inverse variance-weighted multi-SNP meta-analyses (IVW), with 120 significant associations retained, and additionally manually curated to keep associations between chronotype and mental or cardiometabolic health (see Table S2 for SNP characteristics). Chronotype/chronotype and chronotype/sleep duration studies were discarded, leaving 16 potential studies for investigation (Figure 1).

### 2.1. Mendelian Randomization Investigations

Each retained exposure’s data were downloaded from MR-Base via the TwoSampleMR package version 0.5.5 [8]. For each instrumental variable (IV) SNP in the exposure study, the following procedure was performed. First, the strandedness of each GWAS was checked to ensure that at each allele, the minor and major alleles were equal. If these were reversed, effect sizes were modified to correct for this. Pallendromic SNPs, which contain alleles represented by the same base pairs on both strands of DNA, were discarded. If SNPs were not present, proxies were found using PLINK with an $R^{2}$ of at least 0.8, and strand was checked again [10]. Next, SNPs, in the exposure GWAS set, were clumped by LD to ensure statistical independence. In a window of 10,000 base pairs, an $R^{2}$ cutoff of <0.001 was set to obtain haplotype blocks using the European reference panel of the 10,000 Genomes Project [11]. In each exposure/outcome pair, this left a variable number of SNPs for use as valid, independent IVs (see Figure 1 top second step). Effect sizes for each SNP were reported as a β or the transformed log(OR). The Wald ratio was then obtained, giving a measure of the effect of the exposure on the outcome [12]:

(1) $$\hat{\theta_{j}}=\frac{\beta_{Yj}}{\beta_{Xj}}$$

where *β_Y_j* is the effect of the IV on the outcome, and *β_X_j* is the effect of the IV on the exposure is obtained for SNP j, and $\hat{\theta }$ is the effect size.

An initial IVW analysis between each exposure/outcome set was performed, with the false discovery rate (FDR) controlled for [13]. An FDR of <0.05 was considered significant. Rather than calculate Wald ratios individually, the outcome GWAS βs or odds ratios are regressed on the exposure in an inverse variance-weighted (IVW) meta-analysis. The slope of the regression line indicates the strength of the effect, as an increase in the unit of outcome per unit of the exposure [14]. In a IVW meta-analysis, the IVW estimate is calculated by:

(2) $${\hat{\beta }}_{Yj}=\theta_{IVW}{\hat{\beta }}_{Xj}+\epsilon_{Ij};\epsilon_{Ij}∼N\left(0,\sigma^{2}se{\left({\hat{\beta }}_{Yj}\right)}^{2}\right)$$

where $\hat{\theta }$ is the inverse variance-weighted average, se is the standard error, ϵ is an error term (Figure 1 third step, right), and other terms are as above.

Ten significant IVW studies were then subject to four additional MR methods (Figure 1, third level left). To address unseen horizontal pleiotropy, the Egger regression was performed [15,16]. The Wald ratios of each SNP are used in meta-regression by taking the inverse variance weights used in the IVW analysis without modeling the intercept. As a result, a causal estimate, similar to IVW, is obtained, adjusted though for horizontal pleiotropy which is necessary so as to ensure IVW validity [16]. MR-Egger regression is an extension of IVW regression. Instead of assuming no intercept term, an intercept is estimated:

(3) $${\hat{\beta }}_{Yj}=\theta_{0E}+\theta_{1E}{\hat{\beta }}_{Xj}+\epsilon_{Ej};\epsilon_{Ej}∼N\left(0,\sigma^{2}se{\left({\hat{\beta }}_{Yj}\right)}^{2}\right)$$

where $\theta_{0E}$ is the intercept and $\theta_{1E}$ the MR-Egger estimate. If the intercept is equal to zero, then the IVW method and MR-Egger will be equivalent [14]. During the IVW process in MR-Egger, the effect sizes of each SNP must have the same sign, and this decreases the variation between them [16].

Inverse variance-weighted median analyses were performed, using the median of the Wald ratios as an effect size. Unweighted analyses assume that over half of the instruments are valid, while in a weighted median analysis the assumption is that the at least 50% of the weight of the instruments are valid themselves [17]. This approach is robust to directional pleiotropy when compared to a simple IVW meta-analysis.

The mode-based estimator (MBE) clusters Wald ratios before calculating random effects in an IVW meta-analysis [18]. The simple MBE uses unweighted analysis, while the weighted MBE uses inverse variance weighting. First, a smooth empirical density function is calculated for each Wald ratio and are then clustered. The Zero Modal Pleiotropy Assumption states that the biggest cluster with the same ratio estimates will be valid instruments. These were used in each analysis, resulting in fewer SNPs and less power, but an increase in potential robustness to horizontal pleiotropy.

Heterogeneity can occur when individual SNPs do not converge on an estimate; this was estimated by Cochran’s *Q* [19]. In this context, heterogeneity may be a sign of horizontal pleiotropy, wherein SNPs effect the outcome by their influence on other confounding traits [20]. To inspect SNPs for outliers, we performed leave-one-out sensitivity analyses using the IVW method, leaving out one SNP in each analysis.

We used the Steiger test to access the directionality of all causative analyses post hoc [21]. The Steiger test first assesses which variables (exposure or outcome) are influenced by the SNPs used, by testing if the SNPs explain more variance in the exposure than in the outcome with a modified *Z* statistic. If the *p*-value of the IVW estimate and the Steiger estimate are both significant, the sign of the *Z* statistic is used to assign the correct causal direction between exposure and outcome.

### 2.2. Confounder and Intermediate Analysis

To address potential confounders, not seen in pairwise analyses even in the absence of the statistical suggestion of horizontal pleiotropy, existing MR studies were obtained from EpiGraphDB (Figure 1 last step). Each study exposure/outcome pair with significant FDR-corrected IVW results was used to interrogate the database for intermediate, reverse intermediate, collider, and confounding variables. Results from the study database surpassing a *p*-value threshold of <1 × 10^−5^, using a fixed-effects IVW method only, were retained. The β effect sizes for significant results between exposures and outcomes, exposures and confounders, and outcomes and confounders were used to create a weighted directed graph in Cytoscape [22] version 3.8.2, and the yFiles Organic (force directed) layout to visualize the relationships between traits.

All statistical analyses were performed in R version 4.0.5 [23].

## 3. Results

**Figure 2:**
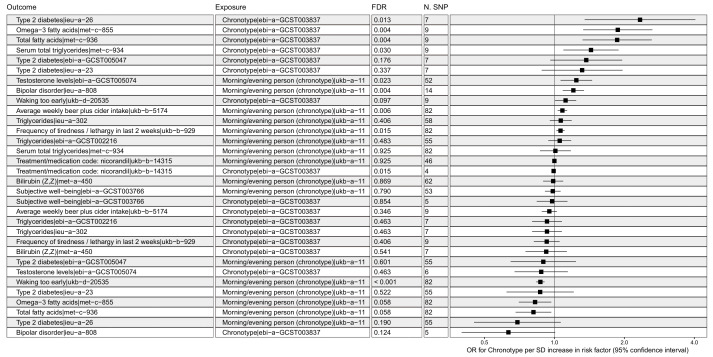
Causal relationships between exposure to chronotype and various traits. Causal associations mined from EpiGraphDB (IVW *p* < 5 × 10^−8^) were analyzed using two chronotype exposure measures. Outcomes are listed with trait and study accession number. Exposure *ebi-a-GCST003837* reflects SD increase in chronotype on a continuous morning–evening scale. Exposure *ukb-a-11* represents odds of an evening chronotype. Effect sizes are from random-effects inverse variance-weighted analyses with 95% confidence intervals. False discovery rate-corrected *p*-values are shown.

Initially, 120 prospective studies were identified using EpiGraphDB containing chronotype as an exposure,. Of these, there were 28 primary associations, with IVW *p*-values of <5 × 10^−8^, directly relevant to this study, including measures of alcohol intake, bipolar disorder, T2DM (type 2 diabetes mellitus) as well as testosterone and triglyceride levels. See Table S1. Since the analyses were performed in a high-throughput environment, we re-analyzed each relevant study with the IVW method using summary statistics (Figure 2). Following filtering and quality control, 10 studies held up to a false discovery rate of *p* < 0.05. The bi-directional nature of the forest plot exists due the different nature of the chronotype exposure studies (treating morning or evening chronotype as case/control). Each of the 10 studies were further analyzed with a tendency toward eveningness reflecting a high β value in the exposure effect size.

### 3.1. Chronotype Influences on Diabetes, Alcohol Consumption, and Bipolar Disorder

**Figure 3:**
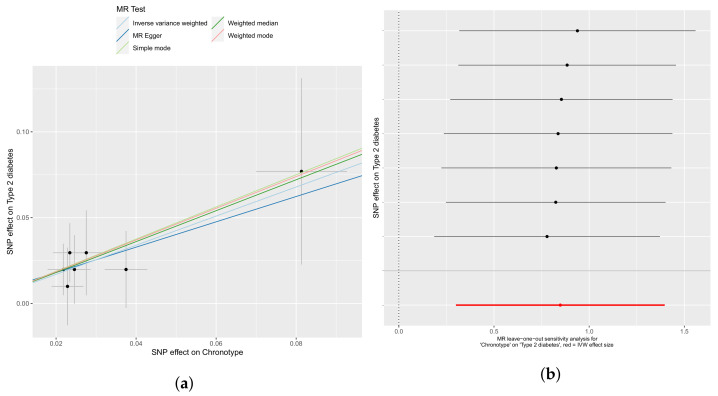
An evening chronotype causes increased odds of a type 2 diabetes mellitus diagnosis. (**a**) IVW, weighted median, mode, weighted mode, and Egger regressions shown. Panel (**b**) depicts leave-one-out sensitivity analyses with the IVW method, where the red line indicates the consensus IVW point estimate.

The strongest associations (β > 0.90) include risk for T2DM and total fatty acid concentration. T2DM included seven independent SNPs, see Table S3. The weakest association was with the MR-Egger method, β = 0.73, which while not highly significant (*p* = 0.04) did not reveal evidence of horizontal pleiotropy (intercept *p*-value = 0.98) or heterogeneity (Egger Q *p*-value 0.99). The IVW analysis and weighted median analysis each concurred (*p* = 0.002 and 0.009), suggesting little loss of associative signal even if some of the seven SNPs were biased by pleiotropy (Figure 3a). Leave-one-out sensitivity analyses suggests that no one IV dominates the model, and all methods have similar effect sizes as judged by slope, as shown in Figure 3b.

**Figure 4:**
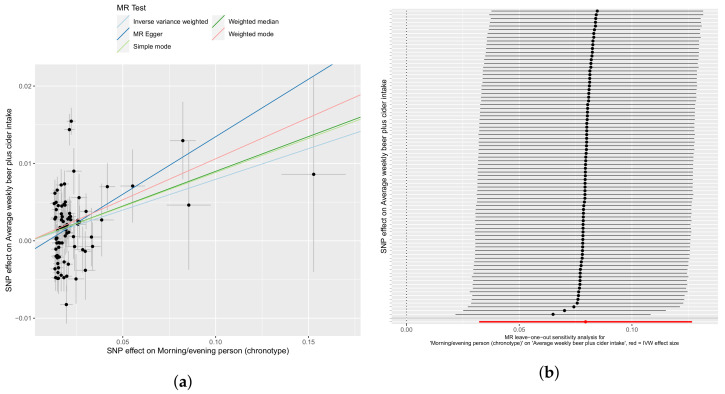
An evening chronotype causes weekly (alcoholic) beer and/or cider intake. (**a**) IVW, weighted median, mode, weighted mode, and Egger regressions shown. Panel (**b**) depicts leave-one-out sensitivity analyses with the IVW method, where the red line indicates the consensus IVW point estimate.

Chronotype has a weaker but more statistically significant association with the amount of alcoholic beer or cider drinks consumed weekly, with the increase reflected in pints per week consumed (mean among all respondents of 3/week). With an MR-Egger intercept *p*-value of 0.31 and a significant Egger regression coefficient (*p*-value = 0.04), the initial analysis provides strong evidence that the trait may not be subject to horizontal pleiotropy. The weighted median and IVW method were again strongest (β = 0.089 and 0.79, *p* = 4.4 × 10^−6^ and 9.7 × 10^−4^), as shown in Figure 4a. There was, however, evidence among the 82 SNPs which passed the threshold for strong heterogeneity (Cochran Q = 333), suggesting pleiotropy or moderating factors for further investigation even in the absence of such suggestions from the MR-Egger intercept significance test. Heterogeneity among SNPs can be observed in Figure 4b, while the leave-one-out analysis shows overlapping confidence intervals in each iteration, suggesting homogeneity on the whole.

**Figure 5:**
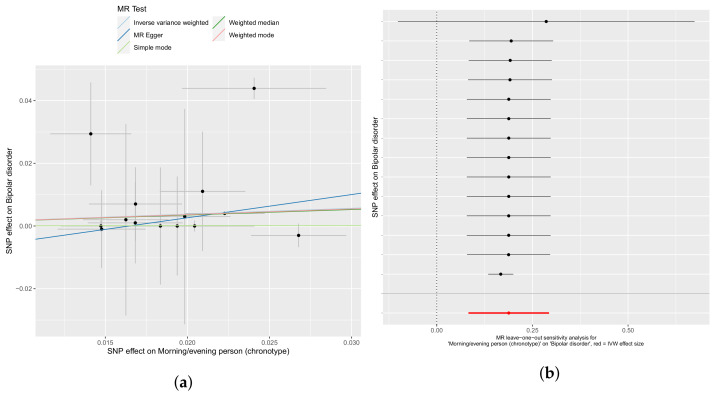
An evening chronotype contributes to the likelihood of a bipolar disorder diagnosis. (**a**) IVW, weighted median, mode, weighted mode, and Egger regressions shown. Panel (**b**) depicts leave-one-out sensitivity analyses with the IVW method, where the red line indicates the consensus IVW point estimate.

The influence of chronotype on bipolar disorder appears substantially varied, with different SNPs with large standard errors apparent in Figure 5. The Cochran’s Q statistic (Q = 149, *p* = 2 × 10^−24^) echoes the visible heterogeneity of SNPs in the analysis. Nevertheless, there is a consistent β effect size among the IVW, weighted median, and weighted mode analyses (0.188, 0.175, and 0.181) with are highly statistically significant (*p* = 4 × 10^−4^, 1 × 10^−11^, and 3 × 10^−6^, respectively). This presents a small but powerful signal that an evening chronotype leads to an increase in activation of pathways leading to bipolar disorder. The test for horizontal pleiotropy, in contrast to the Q statistic, does not suggest horizontal pleiotropy (*p* = 0.47).

Results for other seven individual analyses are given in the Supplemental Materials, Figures S1–S7.

### 3.2. Confounder Case Studies: Bi-Polar Disorder and Alcohol Intake

**Figure 6:**
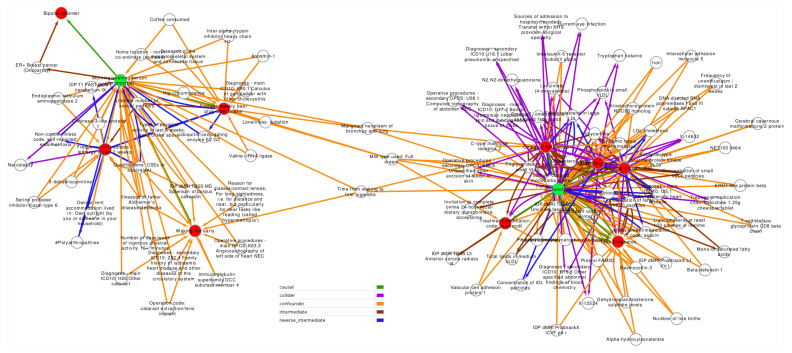
Causal and confounding relationships between chronotype exposures (green nodes) and traits (red nodes). White nodes are potential confounding variables. Edges indicate the direction of causality (arrows). Green edges = causal relationships; purple = colliders, orange = confounders, brown = intermediates, and blue = reverse intermediates.

Each of the 10 exposure/outcome analyses were included in a confounder analysis, searching via existing MR associations for possible intermediate variables. Results in full are available in the Table S4. In Figure 6, a directed graph was created to visually inspect the relationships between types of confounders, outcomes, and potentially causal exposures. Exposures are represented in green, reflecting the two independent GWAS analyses used to access chronotype. Outcomes are reflected in red, and potential intermediate or confounding traits as white notes. As may be evident, there are many potential confounders (111), followed in number by intermediates (52), colliders (35), and reverse intermediates (18) all with *p* < 1 × 10^−5^ in the EpiGraphDB database. The graph shows two clusters sharing potential confounding traits (time to first cigarette and lung cancer). Both are confounders between chronotype and two outcomes: cigarettes confound omega-3 fatty acid concentration and waking too early; lung cancer also confounds omega-3 fatty acid wile also impacting average weekly beer/cider intake.

**Figure 7:**
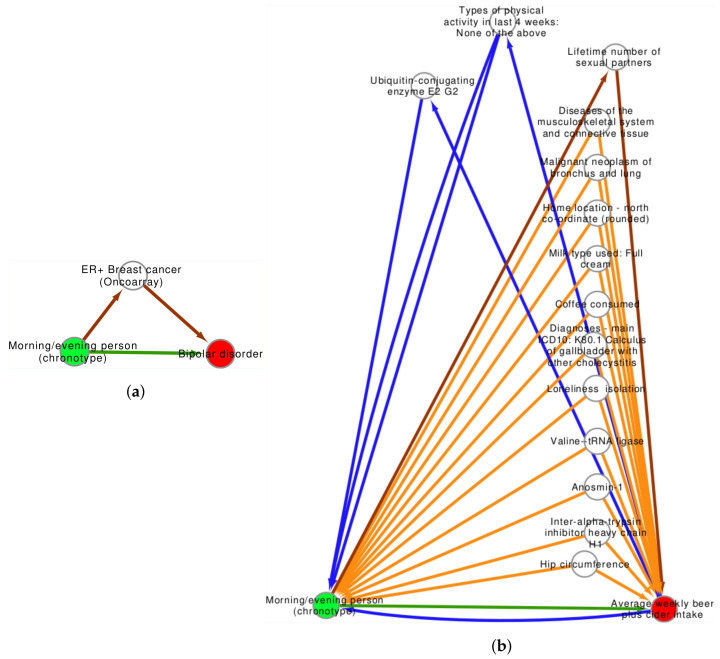
(**a**) An evening chronotype associates with bipolar disorder. In addition to a valid association between chronotype and bipolar disorder, an intermediate association (*p* < 1 × 10^−5^) was found for ER+ breast cancer. (**b**) An evening chronotype associates with increased beer and cider consumption. There is a potential bi-directional relationship between increased alcohol intake and an evening chronotype both directly (blue lines) and mediated by lack of physical activity and elevated UBE2G2. Chronotype may influence the number of sexual partners, which may influence average alcohol intake (brown). There are numerous potential confounders relating body composition, loneliness, and diet.

Not all MR analyses are beset by confounders, however. The relationship between exposure to an evening chronotype and bipolar disorder was not found to be influenced by a statistically significant confounder or collider. In addition to the direct relationship between chronotype and bipolar disorder, a mediator of the odds of ER+ breast cancer was discovered, see Figure 7a. The relationship from chronotype to cancer (β 0.32) was stronger than the unmediated relationship directly to bipolar (β 0.17), but weaker than the possible relationship of breast cancer to bipolar disorder (β 0.42).

As a last example study, the relationship between exposure to an evening chronotype and an increase in beer/wine intake was explored. There is a possible bi-directional relationship between chronotype and beer/alcohol intake, mediated by reverse intermediates of a lack of physical activity (types of physical activity in the last 4 weeks: none of the above) and concentration of the UBE2G2 protein. Additionally, direct intermediates include the lifetime number of sexual partners. No colliders were present, but several potential confounders were identified which may need to be conditioned on, depending on study design. These included dietary consumption (milk type and coffee consumed), loneliness and isolation, hip circumference, and other metabolite measures. See Figure 7b. Data underlying the confounder graph in Figure 6 and Figure 7 are in the Supplementary Table S4.

## 4. Discussion

This analysis characterised the influence of an evening chronotype on cardiometabolic and behavioral traits. Chronotype, a measure of circadian rhythm, is a largely endogenous process though there are sustaining queues, zeitgebers, which influence chronotype [24]. The translational importance of understanding circadian biology is not limited to neurobehavioral function; the interplay between metabolism and circadian biology has been highlighted heavily in recent years. The gut microbiome, adipose cytokines, and metabolic hormones from ghrelin to leptin are all strongly regulated by circadian biology [25,26,27]. Recent Mendelian randomization studies have also suggested a strong causal link between chronotype (a gross circadian phenotype) and body composition, free fatty acid circulation, and adiposity [2,28]. The current analysis revealed strong associations between chronotype and 10 other biological traits. In Figure 2, we reproduced causal associations between diabetes and triglycerides. Additionally, we report novel associations between chronotype and metabolomic data (omega-3 fatty acids, total fatty acids, bilirubin). The strongest signals include diabetes and bipolar disorder, highlighting chronotype as a link between metabolic and psychiatric health. While not all IVW MR studies passed multiple testing correction, those that did had complex relationships with other traits, various degrees of heterogeneity and potential interplay in horizontal pleiotropy. All serum triglyceride associations and a link to type 2 diabetes had the same relationship to chronotype: increased risk or concentration of the trait on an exposure to a genetic predisposition to eveningness. Interestingly, there was a significant (albeit small) association between eveningness and a decrease in likelihood to be taking nicorandil, a ventilator used to treat angina (see Figure S4). Nicorandil has been shown in one study to affect the diurnal rhythms in body temperature and heart rate in rats [29]. There is no evidence that the drug affects circadian behavior in humans, although variant angina (treated with nicorandil) has a circadian component, supporting the idea that the influence of circadian biology may contribute to some types of angina that are amenable to nicorandil treatment [30]. The influence of chronotype on alcohol consumption was recently investigated by Hisler and colleagues, who revealed a 24 hour rhythm of alcohol craving in late adolescent adults and showed that an evening chronotype corresponds to a later craving for alcohol [31]. The associations between an evening chronotype and bipolar disorder are well documented [32], and has been proposed as an endophenotype to classify subtypes of bipolar [33], though several interacting traits may play a role in these subtypes. Not surprisingly, there were over 200 potential confounders or mediators observed between chronotype and the ten significant exposures studied. As evidenced by Figure 6, they vary in type from acceptable in an MR analysis (intermediate, possibly reverse intermediate depending on follow-up studies) to those that must be conditioned upon to maintain causality (the most abundant intermediate, confounders) to the more problematic colliders. Colliders are so called because the causal arrows in a directed acyclic graph from exposure and outcome both impact (collide on) the variable. Unlike confounders, conditioning on colliders in regression introduces bias into the association between exposure and outcome [34]. The four associations subject to collider bias are the relationship between chronotype and omega-3 fatty acids, serum total triglycerides, total fatty acids, and lethargy. Possible confounders for the three metabolites include other cardiometabolic parameters, including total fatty acids, fasting insulin, and VLDL concentrations. The release of metabolomic data in the UK Biobank [35] caters for new opportunities to investigate these collisions experimentally since they relate to chronotype and both psychiatric and cardiometabolic diseases. The only collider associated to lethargy was narcolepsy, an intuitive yet potentially complex relationship. The only confounder between an evening chronotype found in this study was ER+ breast cancer. The confounding was as an intermediate, which maintains the causal nature of the analysis while adding an extra explanatory “path” to get from exposure to outcome. While there is no current literature directly linking chronotype to breast cancer and then to the presence of bipolar disorder, this may be investigated in current murine models of breast cancer via knockdown experiments or by manipulating light as an external zeitgeber.

Instrumental variable analysis relies on strong assumptions, and occasionally unverifiable conditions [36]. Subject knowledge is the most reliable method for concluding assumptions are valid, especially when choosing exposures and outcomes correctly. A naive, phenome-wide search for causation between traits is likely to include among many tests non-plausible experimental designs, leading to an artificial increase in multiple testing burden and design flaws. This is evident by the need to prune associations in the first step of this study. In our re-analysis of traits in step 2, we failed to replicate the extremely low *p*-values present in the EpiGraphDB, even when using the same outcome and exposure studies obtained by a sister database, MR-Base. Differences may be partly explained by analysis choice in the IVW models. We modeled a random effect, assuming that while each SNP contributed overall to the model in a similar direction or magnitude, the Wald ratio generated by each SNP will not be identical. In other words, if each SNP is analogous to a “study” in a meta-analysis, the effect sizes and variances may be similar but a model would not assume they are identical. As a large focus of this study was investigating possible confounders between traits and the possibility of pleiotropy among SNPs, a more conservative random effects approach was chosen as the basis for further analyses. Additionally, the EipGraphDB calculated several models and filtering strategies, and an optimal method for each MR analysis was chosen from a random forest model optimized on artificial data. When re-analyzing data, we chose one strategy, though we included any of their fixed-effect models when incorporating pre-calculated confounder MR analyses as this was the most abundant analysis method in the database. Without access to the underlying filtering methods used in the large repositories, we can speculate that instrumental variable selection, LD clumping, and filtering also played a part. However, this re-analysis has produced associations that stand up to multiple testing while revealing novel associations. This study was limited to viewing causality through the lens of Mendelian randomization alone. In our previous work, we have used gene expression data to identify causal regulatory networks using Bayesian approaches [37]. Opportunities exist to combine these approaches, creating multimodal graphs of gene regulatory networks from that approach with the multitrait networks created in this work.

## 5. Conclusions

Our findings have revealed potential causal associations between chronotype and several behavioral and metabolic traits. While using publicly available data to confirm previously known associations (T2DM, cholesterol) we have found novel associations which may be validated experimentally in models. These include associations between chronotype and the vasodilator nicorandil, and a relationship between chronotype and bipolar disorder potentially mediated by estrogen-receptive breast cancer. Lastly, we have revealed potential confounders and colliders impacting the relationship between chronotype and commonly reported cardiometabolic traits which should be addressed through a combination of multivariate and multistage analyses as appropriate.

## Data Availability

Data are available via the EpiGraphDB and MRBase APIs. Data used to produce all figures (including supplementary figures) are available in Tables S1–S4.

## Supplementary Materials

The following are available online at https://www.mdpi.com/article/10.3390/genes12071029/s1, Table S1: EpiGraphDB data downloaded to produce Figure 2. Table S2: SNP-level information on all 10 studies analyzed needed to reproduce analyses of these 10 studies. Table S3: Summary level results for each of the 10 MR analyses. Table S4: Statistics downloaded from EpiGraphDB needed to reproduce Figure 6 and Figure 7. Figures S1–S7: Remaining MR Results Figures.

## Abbreviations

The following abbreviations are used in this manuscript:

GWAS	Genome-wide association study
MR	Mendelian randomization
IVW	Inverse-variance weighted
OR	Odds ratio
SNP	Single nucleotide polymorphism
LD	linkage disequilibrium
IV	Instrumental variable
MBE	mode-based estimator
FDR	alse discovery rate
T2DM	Type 2 diabetes melletus
ER+	Estrogen Receptor positive
VLDL	Very low density lipoprotein

## References

[1] Lind M.J., Brick L.A., Gehrman P.R., Duncan L.E., Gelaye B., Maihofer A.X., Nievergelt C.M., Nugent N.R., Stein M.B., Amstadter A.B. (2020). Psychiatric Genomics Consortium Posttraumatic Stress Disorder. Molecular genetic overlap between posttraumatic stress disorder and sleep phenotypes. Sleep.

[2] Adams C.D., Neuhausen S.L. (2019). Evaluating causal associations between chronotype and fatty acids and between fatty acids and type 2 diabetes: A Mendelian randomization study. Nutr. Metab. Cardiovasc. Dis. NMCD.

[3] Richmond R.C., Anderson E.L., Dashti H.S., Jones S.E., Lane J.M., Strand L.B., Brumpton B., Rutter M.K., Wood A.R., Straif K. (2019). Investigating causal relations between sleep traits and risk of breast cancer in women: Mendelian randomisation study. BMJ (Clin. Res. Ed.).

[4] Gibson M., Munafò M.R., Taylor A.E., Treur J.L. (2019). Evidence for Genetic Correlations and Bidirectional, Causal Effects Between Smoking and Sleep Behaviors. Nicotine Tob. Res. Off. J. Soc. Res. Nicotine Tob..

[5] Treur J.L., Gibson M., Taylor A.E., Rogers P.J., Munafò M.R. (2018). Investigating genetic correlations and causal effects between caffeine consumption and sleep behaviours. J. Sleep Res..

[6] Lane J.M., Vlasac I., Anderson S.G., Kyle S.D., Dixon W.G., Bechtold D.A., Gill S., Little M.A., Luik A., Loudon A. (2016). Genome-wide association analysis identifies novel loci for chronotype in 100,420 individuals from the UK Biobank. Nat. Commun..

[7] Buniello A., MacArthur J.A.L., Cerezo M., Harris L.W., Hayhurst J., Malangone C., McMahon A., Morales J., Mountjoy E., Sollis E. (2019). The NHGRI-EBI GWAS Catalog of published genome-wide association studies, targeted arrays and summary statistics 2019. Nucleic Acids Res..

[8] Hemani G., Zheng J., Elsworth B., Wade K.H., Haberland V., Baird D., Laurin C., Burgess S., Bowden J., Langdon R. (2018). The MR-Base platform supports systematic causal inference across the human phenome. eLife.

[9] Liu Y., Elsworth B., Erola P., Haberland V., Hemani G., Lyon M., Zheng J., Lloyd O., Vabistsevits M., Gaunt T.R. (2020). EpiGraphDB: A database and data mining platform for health data science. Bioinformatics.

[10] Chang C.C., Chow C.C., Tellier L.C., Vattikuti S., Purcell S.M., Lee J.J. (2015). Second-generation PLINK: Rising to the challenge of larger and richer datasets. GigaScience.

[11] Huang J., Howie B., McCarthy S., Memari Y., Walter K., Min J.L., Danecek P., Malerba G., Trabetti E., Zheng H.F. (2015). Improved imputation of low-frequency and rare variants using the UK10K haplotype reference panel. Nat. Commun..

[12] Rasooly D., Patel C.J. (2019). Conducting a Reproducible Mendelian Randomization Analysis Using the R Analytic Statistical Environment. Curr. Protoc. Hum. Genet..

[13] Benyamin B., Visscher P.M., McRae A.F. (2009). Family-based genome-wide association studies. Pharmacogenomics.

[14] Burgess S., Thompson S.G. (2017). Interpreting findings from Mendelian randomization using the MR-Egger method. Eur. J. Epidemiol..

[15] Bowden J., Del Greco M.F., Minelli C., Davey Smith G., Sheehan N., Thompson J. (2017). A framework for the investigation of pleiotropy in two-sample summary data Mendelian randomization. Stat. Med..

[16] Bowden J., Davey Smith G., Burgess S. (2015). Mendelian randomization with invalid instruments: Effect estimation and bias detection through Egger regression. Int. J. Epidemiol..

[17] Bowden J., Davey Smith G., Haycock P.C., Burgess S. (2016). Consistent Estimation in Mendelian Randomization with Some Invalid Instruments Using a Weighted Median Estimator. Genet. Epidemiol..

[18] Hartwig F.P., Davey Smith G., Bowden J. (2017). Robust inference in summary data Mendelian randomization via the zero modal pleiotropy assumption. Int. J. Epidemiol..

[19] Higgins J., Green S. Cochrane Handbook for Systematic Reviews of Interventions, 5.1.0 [updated march 2011] ed.; The Coochrane Collaboration: 2011. www.training.cochrane.org/handbook.

[20] Burgess S., Small D.S., Thompson S.G. (2017). A review of instrumental variable estimators for Mendelian randomization. Stat. Methods Med. Res..

[21] Hemani G., Tilling K., Davey Smith G. (2017). Orienting the causal relationship between imprecisely measured traits using GWAS summary data. PLoS Genet..

[22] Shannon P., Markiel A., Ozier O., Baliga N.S., Wang J.T., Ramage D., Amin N., Schwikowski B., Ideker T. (2003). Cytoscape: A software environment for integrated models of biomolecular interaction networks. Genome Res..

[23] R Core Team (2013). R: A Language and Environment for Statistical Computing.

[24] Albrecht U. (2012). Timing to perfection: The biology of central and peripheral circadian clocks. Neuron.

[25] Li Y., Ma J., Yao K., Su W., Tan B., Wu X., Huang X., Li T., Yin Y., Tosini G. (2020). Circadian Rhythms and Obesity: Timekeeping Governs Lipid Metabolism. J. Pineal Res..

[26] Socaciu A.I., Ionuţ R., Socaciu M.A., Ungur A.P., Bârsan M., Chiorean A., Socaciu C., Râjnoveanu A.G. (2020). Melatonin, an ubiquitous metabolic regulator: Functions, mechanisms and effects on circadian disruption and degenerative diseases. Rev. Endocr. Metab. Disord..

[27] Pan X., Mota S., Zhang B. (2020). Circadian Clock Regulation on Lipid Metabolism and Metabolic Diseases. Adv. Exp. Med. Biol..

[28] Jones S.E., Lane J.M., Wood A.R., van Hees V.T., Tyrrell J., Beaumont R.N., Jeffries A.R., Dashti H.S., Hillsdon M., Ruth K.S. (2019). Genome-wide association analyses of chronotype in 697,828 individuals provides insights into circadian rhythms. Nat. Commun..

[29] Gantenbein M., Attolini L., Bruguerolle B. (1998). Nicorandil affects diurnal rhythms of body temperature, heart rate and locomotor activity in rats. Eur. J. Pharmacol..

[30] Kusama Y., Kodani E., Nakagomi A., Otsuka T., Atarashi H., Kishida H., Mizuno K. (2011). Variant angina and coronary artery spasm: The clinical spectrum, pathophysiology, and management. J. Nippon. Med. Sch. Nippon Ika Daigaku Zasshi.

[31] Hisler G.C., Rothenberger S.D., Clark D.B., Hasler B.P. (2021). Is there a 24-hour rhythm in alcohol craving and does it vary by sleep/circadian timing?. Chronobiol. Int..

[32] Ferrer A., Costas J., Gratacos M., Martínez-Amorós È., Labad J., Soriano-Mas C., Palao D., Menchón J.M., Crespo J.M., Urretavizcaya M. (2020). Clock gene polygenic risk score and seasonality in major depressive disorder and bipolar disorder. Genes Brain Behav..

[33] Romo-Nava F., Blom T.J., Cuellar-Barboza A.B., Winham S.J., Colby C.L., Nunez N.A., Biernacka J.M., Frye M.A., McElroy S.L. (2020). Evening chronotype as a discrete clinical subphenotype in bipolar disorder. J. Affect. Disord..

[34] Rohrer J.M. (2018). Thinking Clearly About Correlations and Causation: Graphical Causal Models for Observational Data. Adv. Methods Pract. Psychol. Sci..

[35] Allen N.E., Sudlow C., Peakman T., Collins R., Biobank O.b.O.U. (2014). UK Biobank Data: Come and Get It. Sci. Transl. Med..

[36] Labrecque J., Swanson S.A. (2018). Understanding the Assumptions Underlying Instrumental Variable Analyses: A Brief Review of Falsification Strategies and Related Tools. Curr. Epidemiol. Rep..

[37] Aziz F., Acharjee A., Williams J.A., Russ D., Bravo-Merodio L., Gkoutos G.V. (2020). Biomarker prioritisation and power estimation using ensemble gene regulatory network inference. Int. J. Mol. Sci..

