# Supplementary material for: A Causal Web between Chronotype and Metabolic Health Traits
Source: Genes (Basel). 2021 Jul 1;12(7):1029. doi: 10.3390/genes12071029 (PMC8303793; doi:10.3390/genes12071029)
Supplement: Supplementary file 1 [file genes-12-01029-s001.zip › Supplementary/FiguresS1-S7.pdf]

Supplementary Material:
A causal web between chronotype and metabolic health traits

Table S1: TableS1EpigraphDBIVWresults.xlsx. EpiGraphDB data downloaded to produce Figure 2.

Table S2: TableS2StudyCharacteristics.xlsx. SNP-level information on all 10 studies analyzed needed to reproduce analyses of these 10 studies.

Table S3: TableS3MRAnalysisResults.xlsx. Summary level results for each of the 10 MR analyses, including leave-one-out statistics, MR-Egger statistics, and all MR statistics. Used to produce supplementary scatter plots, leave-one-out plots, and Figures 3-5 and supplementary figures.

Table S4: TableS4ConfounderGraph.xlsx. Statistics downloaded from EpiGraphDB needed to reproduce figures 6 and 7.

Figures S1-S7: Following Pages

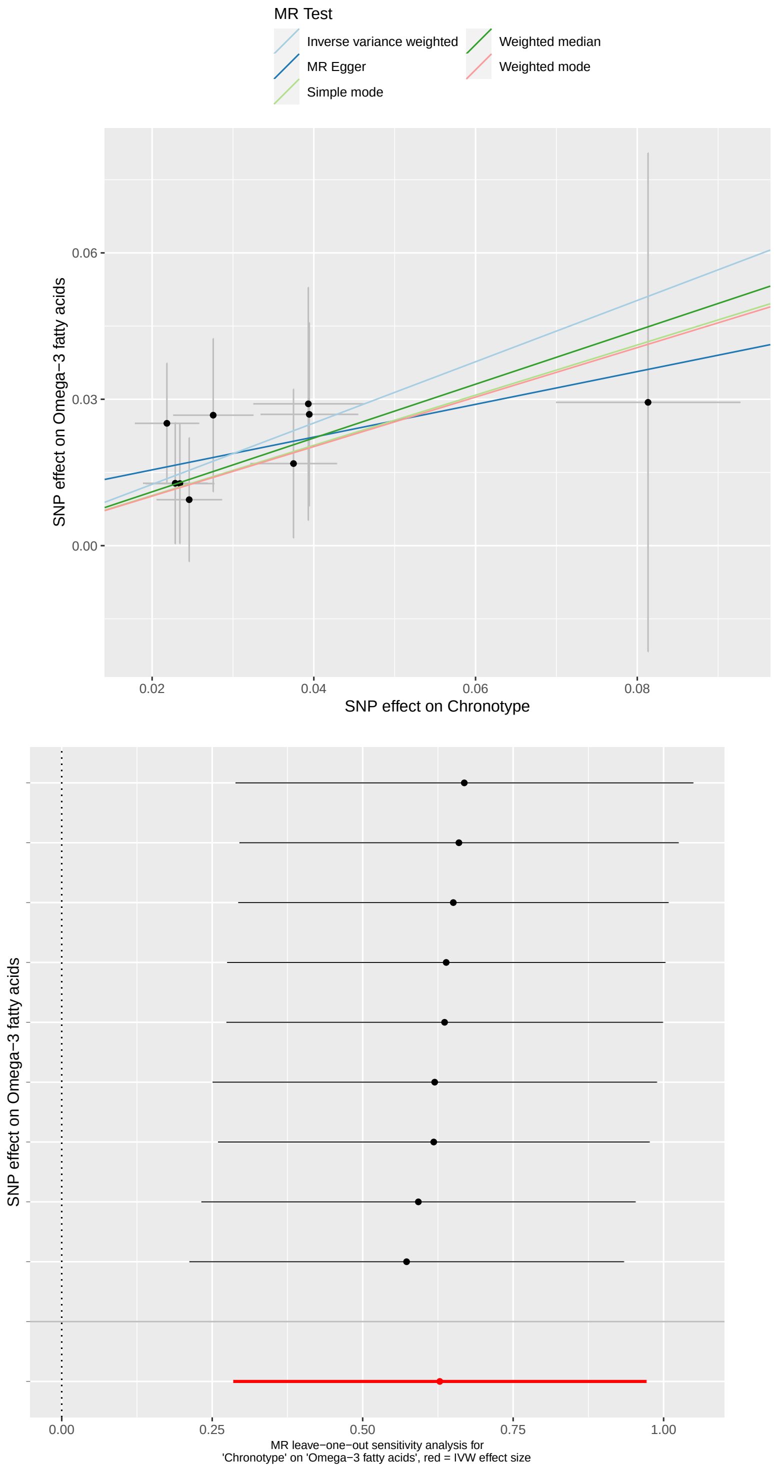

Figure S1: Propensity to an evening chronotype causes increased omega-3 fatty acids. (top) IVW, Weighted median, mode, weighted mode, and Egger regressions shown. Panel (bottom) depicts leave-one-out sensitivity analyses with the IVW method, where the red line indicates the consensus IVW point estimate.

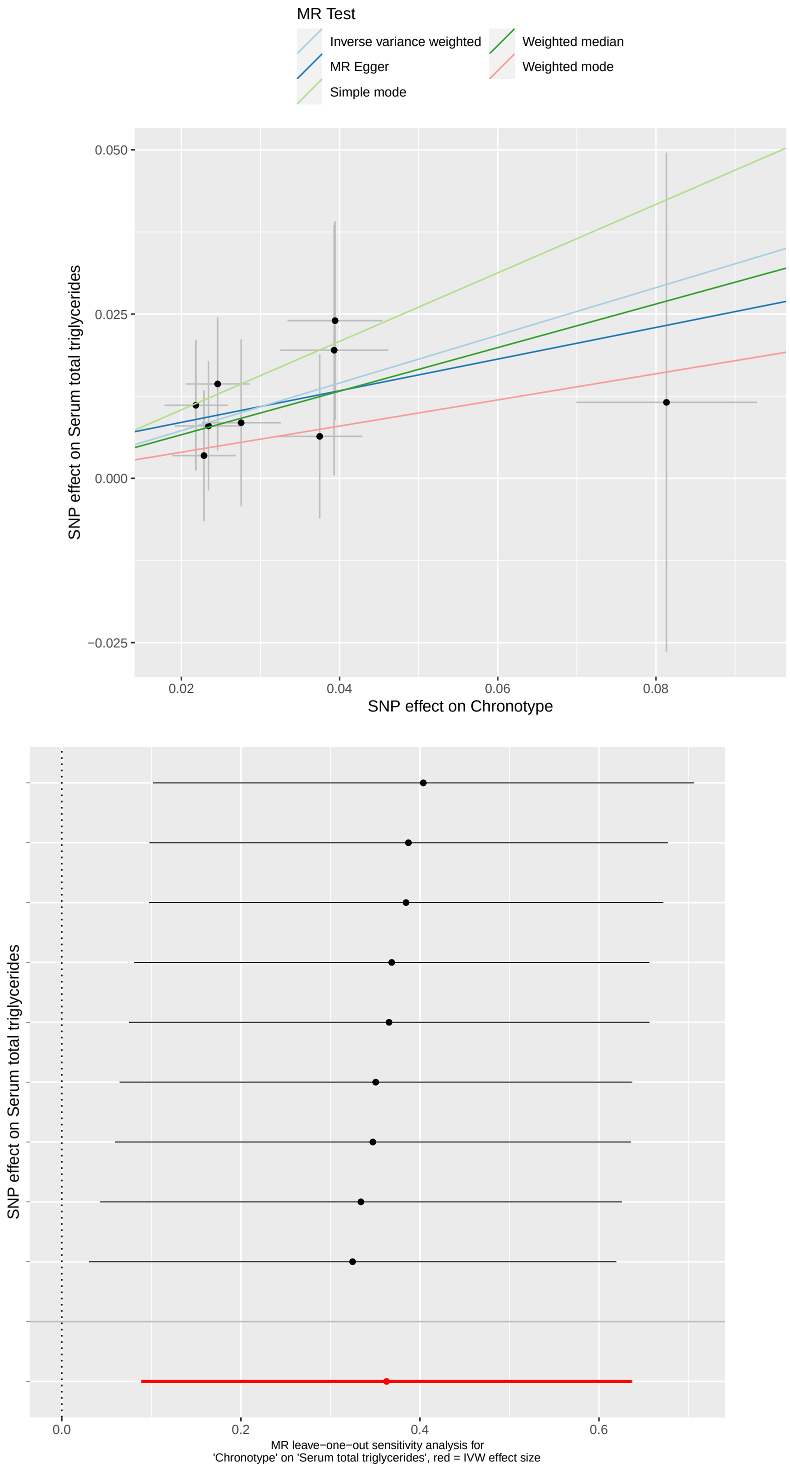

Figure S2: Propensity to an evening chronotype causes increased serum total triglycerides. (top) IVW, Weighted median, mode, weighted mode, and Egger regressions shown. Panel (bottom) depicts leave-one-out sensitivity analyses with the IVW method, where the red line indicates the consensus IVW point estimate.

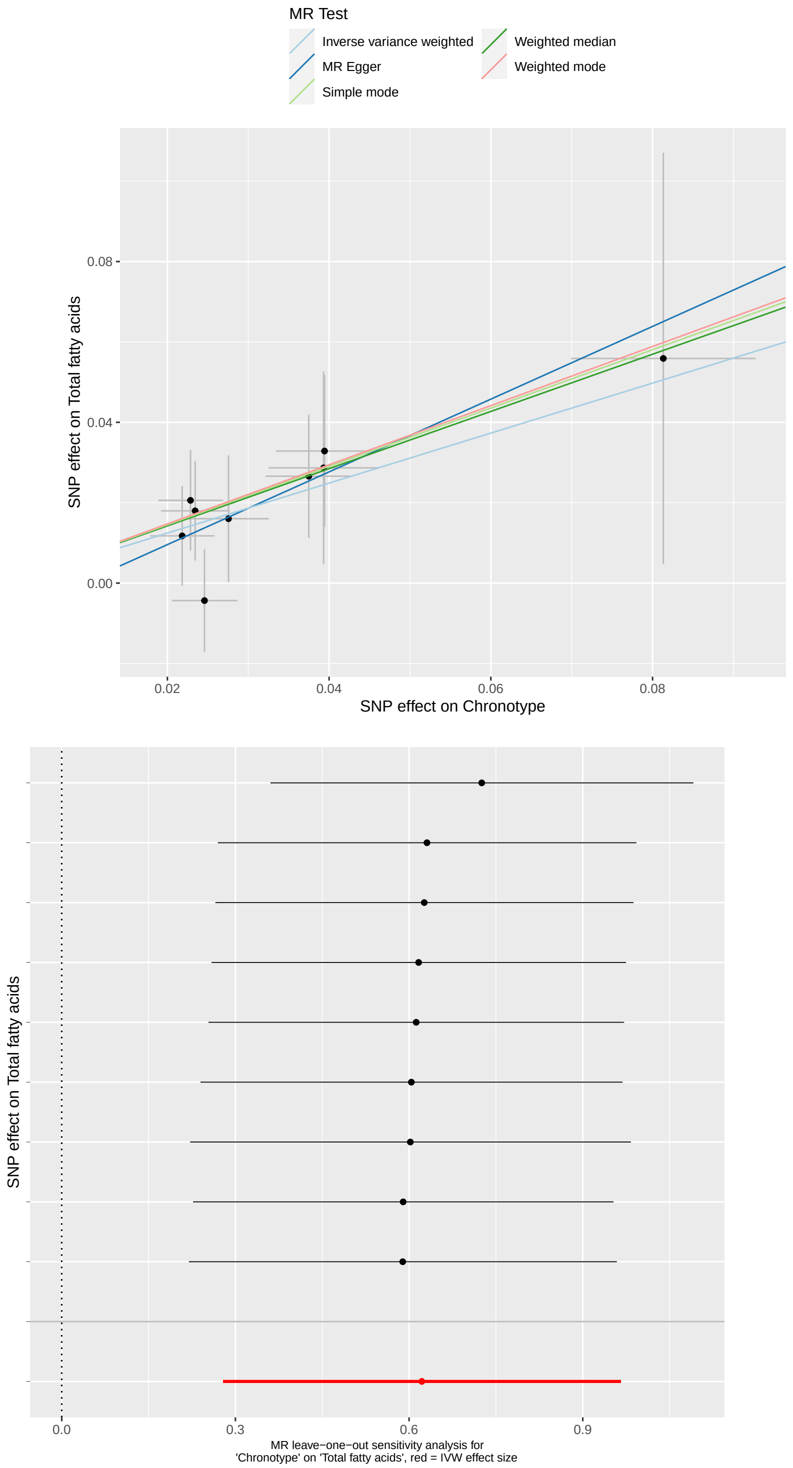

Figure S3: Propensity to an evening chronotype causes increased total fatty acids. (top) IVW, Weighted median, mode, weighted mode, and Egger regressions shown. Panel (bottom) depicts leave-one-out sensitivity analyses with the IVW method, where the red line indicates the consensus IVW point estimate.

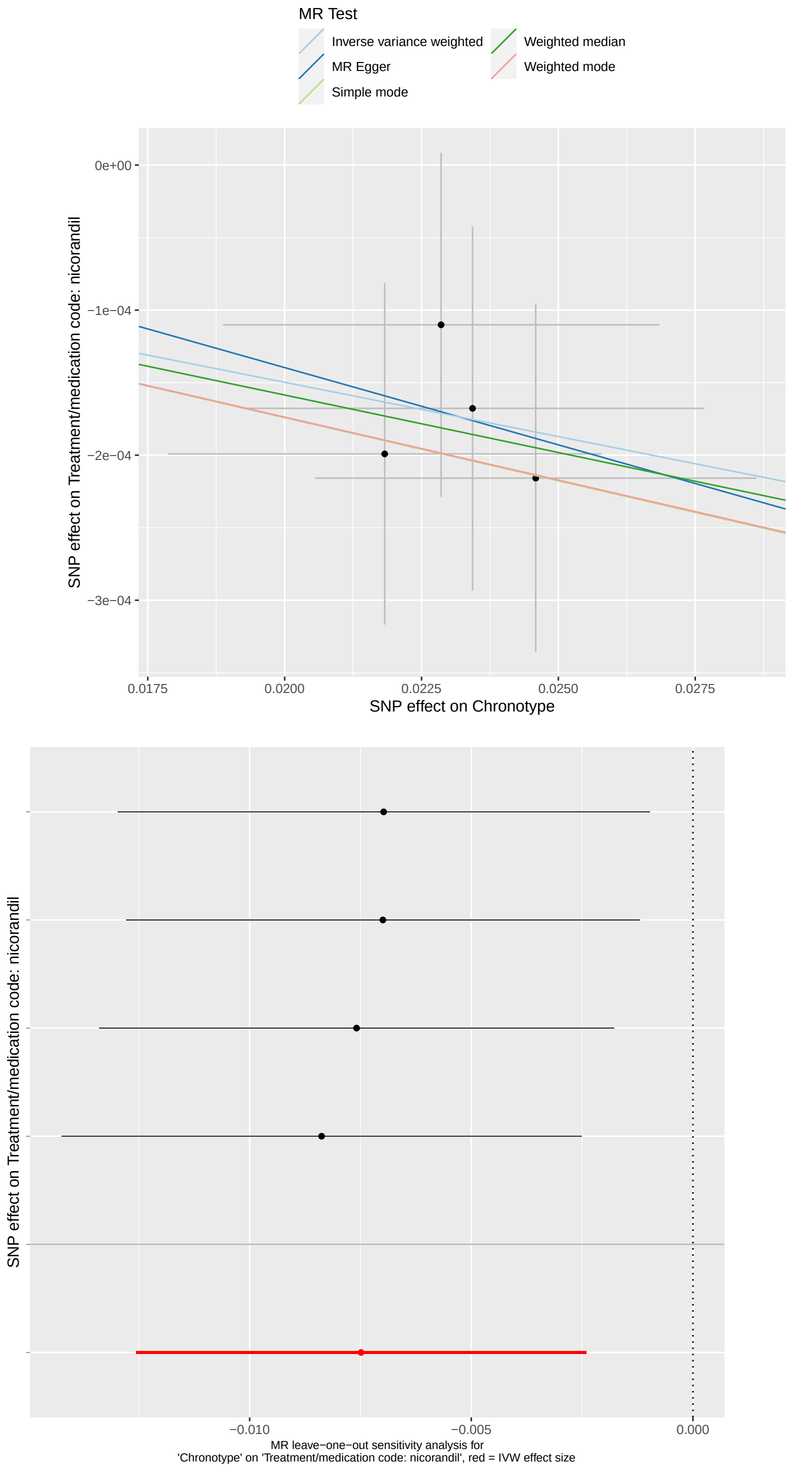

Figure S4: Propensity to an evening chronotype causes decreased likelihood of treatment with nicorandil. (top) IVW, Weighted median, mode, weighted mode, and Egger regressions shown. Panel (bottom) depicts leave-one-out sensitivity analyses with the IVW method, where the red line indicates the consensus IVW point estimate.

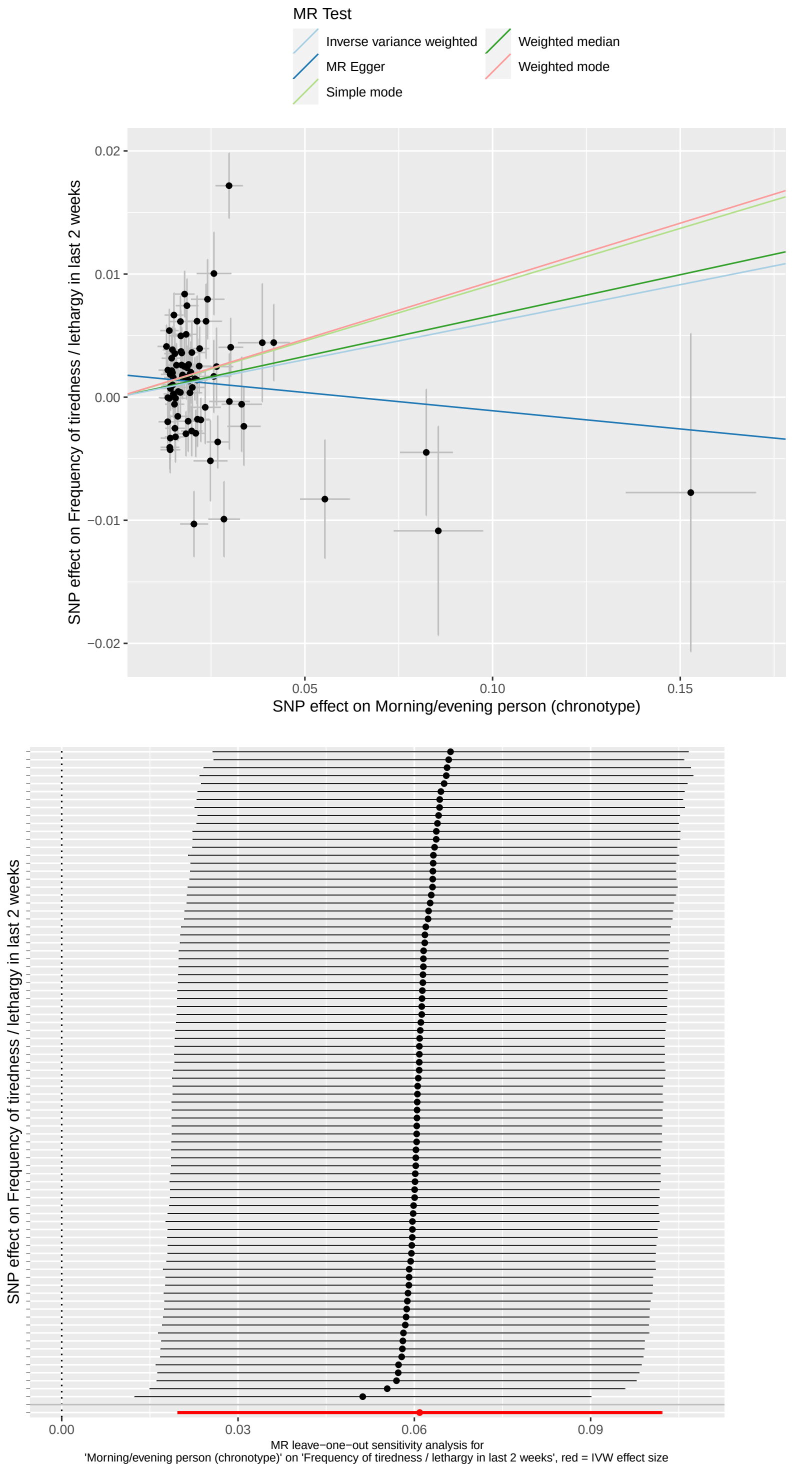

Figure S5: Propensity to an evening chronotype causes increases in self-reported lethargy in the last two weeks. (top) IVW, Weighted median, mode, weighted mode, and Egger regressions shown. Panel (bottom) depicts leave-one-out sensitivity analyses with the IVW method, where the red line indicates the consensus IVW point estimate.

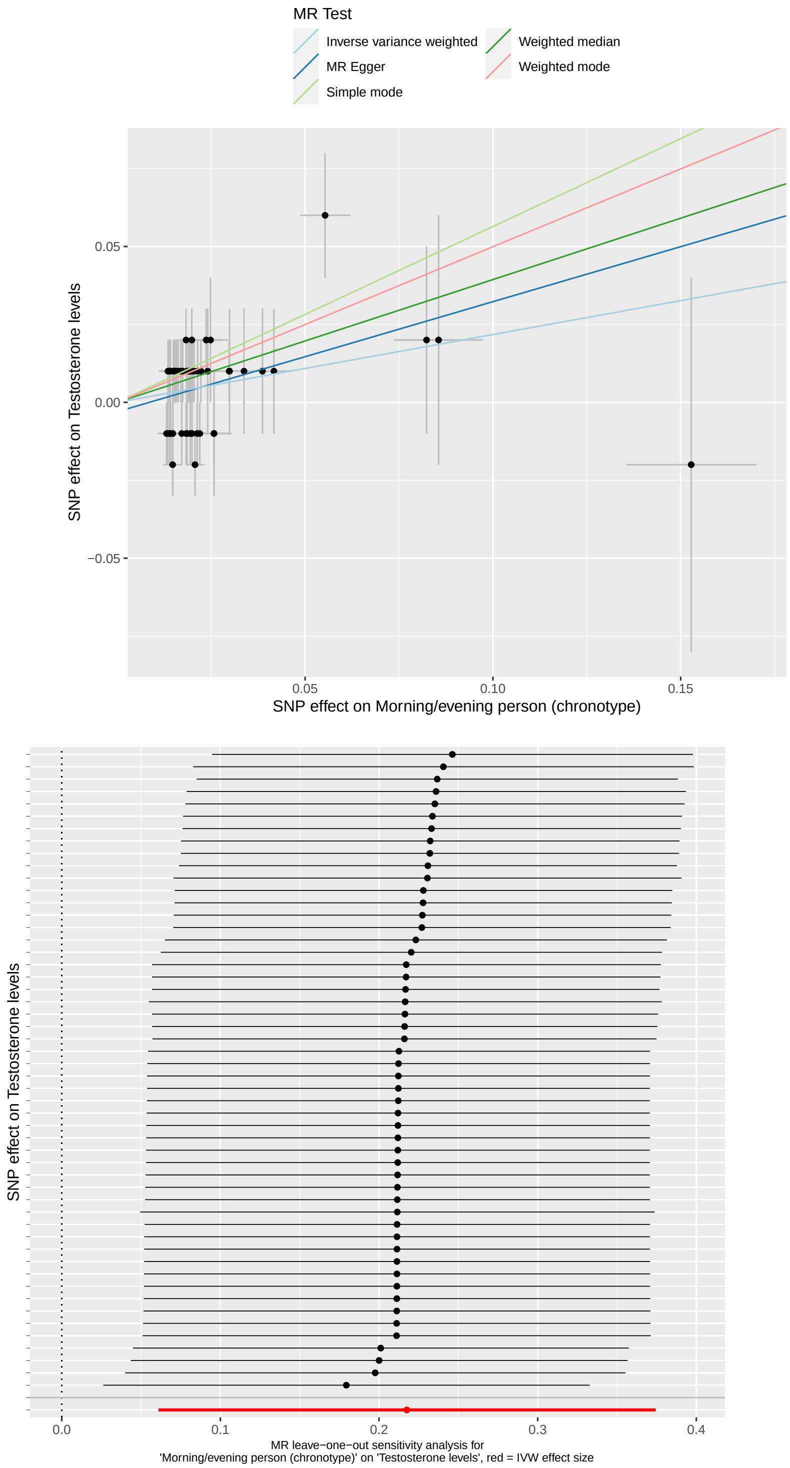

Figure S6: Propensity to an evening chronotype causes increases in serum testosterone. (top) IVW, Weighted median, mode, weighted mode, and Egger regressions shown. Panel (bottom) depicts leave-one-out sensitivity analyses with the IVW method, where the red line indicates the consensus IVW point estimate.

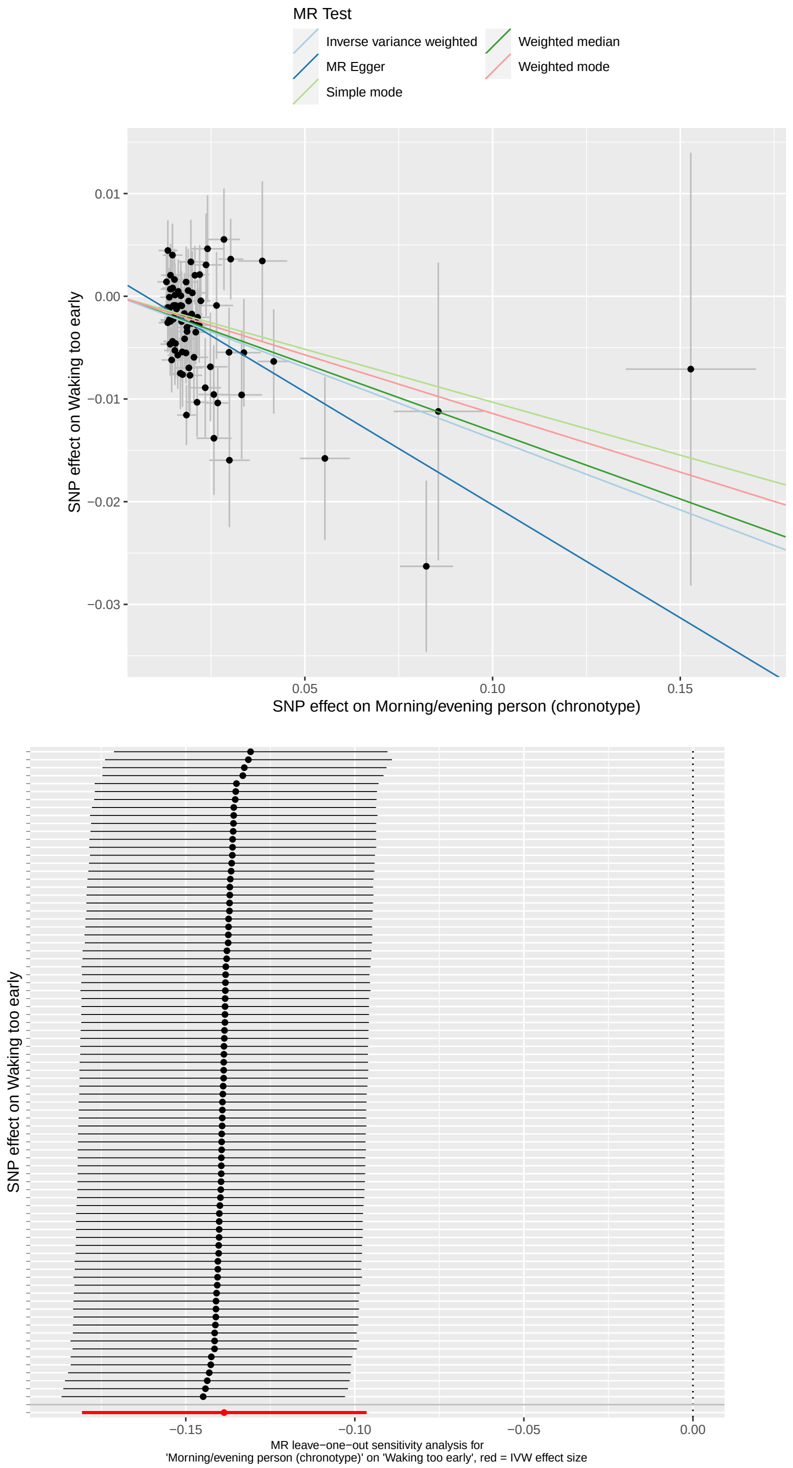

Figure S7: Propensity to an evening chronotype causes decrease in odds of waking early. (top) IVW, Weighted median, mode, weighted mode, and Egger regressions shown. Panel (bottom) depicts leave-one-out sensitivity analyses with the IVW method, where the red line indicates the consensus IVW point estimate.
